# RETRACTED: Iacono, D.; Feltis, G.C. Idea Density and Grammatical Complexity as Neurocognitive Markers. *Brain Sci.* 2025, *15*, 1022

**DOI:** 10.3390/brainsci16040393

**Published:** 2026-04-03

**Authors:** Diego Iacono, Gloria C. Feltis

**Affiliations:** 1 Neuropathology Research, Biomedical Research Institute of New Jersey (BRInj), 140 East Hanover Avenue, Cedar Knolls, NJ 07927, USA; 2 Neuroscience Research, MidAtlantic Neonatology Associates (MANA), Department of Pediatrics, Atlantic Health System (AHS), Morristown, NJ 07927, USA; 3 Neurodevelopmental Research Lab, Biomedical Research Institute of New Jersey (BRInj), Cedar Knolls, NJ 07927, USA; 4 Department of Neurology, Thomas Jefferson University, Philadelphia, PA 07927, USA; 5 Independent Researcher, Bethesda, MD 20817, USA; gloria.libinfoscience@gmail.com

The journal retracts the article titled “Idea Density and Grammatical Complexity as Neurocognitive Markers” [1], cited above.

Following publication, concerns were brought to the attention of the Editorial Office regarding the presence of misnamed phrases and the accuracy of several references cited in this paper [1].

In accordance with the journal’s standard procedures, the Editorial Office and Editorial Board conducted an investigation that confirmed the presence of a misnamed phrase for ROSE model and significant number of non-existent references, which were determined to undermine the overall reliability of this study. In addition, the investigation identified indications of generative AI use in the preparation of the manuscript, without appropriate transparency or discloser, as required by MDPI’s GenAI policy. Although the authors cooperated with the investigation, the explanations and supporting materials provided were not deemed sufficient by the Editorial Board to resolve the identified concerns. As a result, the Editorial Board has lost confidence in the reliability of the findings and decided to retract this publication [1], as per MDPI’s retraction policy.

This retraction was approved by the Editor-in-Chief of the *Brain Sciences* journal.

The authors have been informed of this retraction and have indicated their disagreement with this decision.
